# Genetic Manipulation of Ticks: A Paradigm Shift in Tick and Tick-Borne Diseases Research

**DOI:** 10.3389/fcimb.2021.678037

**Published:** 2021-05-10

**Authors:** Andrew Nuss, Arvind Sharma, Monika Gulia-Nuss

**Affiliations:** ^1^ Department of Biochemistry and Molecular Biology, The University of Nevada, Reno, NV, United States; ^2^ Department of Agriculture, Veterinary, and Rangeland Sciences, The University of Nevada, Reno, NV, United States

**Keywords:** ticks, *Ixodes scapularis*, gene-editing, embryo-injection, CRISPR/Cas-9

## Abstract

Ticks are obligate hematophagous arthropods that are distributed worldwide and are one of the most important vectors of pathogens affecting humans and animals. Despite the growing burden of tick-borne diseases, research on ticks has lagged behind other arthropod vectors, such as mosquitoes. This is largely because of challenges in applying functional genomics and genetic tools to the idiosyncrasies unique to tick biology, particularly techniques for stable genetic transformations. CRISPR-Cas9 is transforming non-model organism research; however, successful germline editing has yet to be accomplished in ticks. Here, we review the ancillary methods needed for transgenic tick development and the use of CRISPR/Cas9, the most promising gene-editing approach, for tick genetic transformation.

## Introduction

Ticks and tick-borne diseases affect animal and human health worldwide and are the cause of significant economic losses. For instance, Lyme disease alone costs approximately $1.3 billion each year in direct medical costs in the United States (Tick-borne Disease Working Group). The tick life cycle begins with an egg containing the developing embryo that hatches into a larva. As the tick proceeds through larval and nymphal stages, a single blood meal is required at each stage, and a final large blood meal by the adult female is needed to develop an egg mass to complete the cycle. This life cycle differs vastly from hematophagous insects, where usually only the adults—and often only females—feed on vertebrate blood, and therefore only adults can vector diseases from infected animals. In contrast, ticks are obligate blood-feeders at all stages of their life cycle, making them viable to transmit pathogens at various life stages. Ticks can transmit many pathogens: bacteria, viruses, protozoans, and fungi (Jongejan and Uilenberg, 2004; Rochlin and Toledo, 2020). *Borrelia* spirochetes, the causative agents of Lyme disease, are among the most important pathogens transmitted by *Ixodes* ticks. However, several other tick-transmitted pathogens are of importance to human and animal health (Eisen and Eisen, 2018). Furthermore, as the tick feeds for extended periods (3-10 days), it interacts with its vertebrate host and can suppress the host’s immune system. In addition to being a vector for pathogens, ticks can cause significant harm to their host due to feeding for a prolonged time: exsanguination when tick infestation is high, secondary infection at the bite site (Eisen and Eisen, 2018), tick paralysis when feeding occurs near the spinal cord (Pienaar et al., 2018), and reactions to tick bites such as alpha-gal syndrome (Commins and Platts-Mills, 2013; Steinke et al., 2015) which induces an allergy to red meat. Understanding tick biology, therefore, is an important research focus.

The process for acaricide discovery and drug/vaccine development against ticks or tick-borne pathogens heavily relies on correlating tick genotype to phenotype. To study the connection of genotype to phenotype, it is important to disrupt gene function and analyze phenotypic effect. Researchers can experimentally regulate gene expression and investigate gene function either at the translational level or at the genetic level using two main biological tools: RNA interference (RNAi) and gene knockouts using editing methods, respectively. CRISPR/Cas9 has emerged as the most straightforward gene editing technology, in comparison to other techniques such as TALENs and ZFNs etc. because of the lower cost and ease of use. Current functional genomics research in ticks primarily depends on RNAi for gene knockdown studies (Karim et al., 2008). However, RNAi has limitations such as incomplete silencing that can vary by target gene, developmental stage, or tissue. In addition, temporary (transient) transcript knockdown limits many studies to a narrow assay window and currently puts transstadial pathogen transmission studies out of reach. Furthermore, RNAi is not suitable for overexpression and transcript rescue experiments required to study functions of the genes that are downregulated, for instance due to pathogen infection. Moreover, RNAi is mostly applicable to adult and sometimes nymphal stages, making gene function studies in embryos and larvae challenging. Still, the reversible nature of knockdowns by RNAi makes it possible to verify the phenotypic effect by restoring protein expression to normal in the same cells. In addition, it may permit knockdown of genes in later life stages that otherwise would result in irreversible developmental abnormalities if knocked down in earlier life stages.

In contrast to RNAi, the advantages of genomic knockouts are absolute silencing, completely blocking protein expression, eliminating any confounding effects from remnant low levels of protein expression post knockdown. Targeted gene knockout and knock-in approaches are therefore highly desirable to investigate tick gene functions that are unanswerable by RNAi alone. The CRISPR/Cas genetic manipulation system is revolutionizing the field of biology, including entomology, by enabling the genetic transformation of diverse arthropods (Bassett and Liu, 2014; Ma et al., 2014; Gilles et al., 2015; Kohno et al., 2016; Li et al., 2017b; Sun et al., 2017) and offers enormous promise for tick research. However, these evolutionarily distinct hematophagous arthropods have quite different embryonic development and life histories than insects (Santos et al., 2013). Germline transformation methods that work for insects may require optimization for ticks. Recently, CRISPR-Cas was used to edit a mite, *Tetranychus urticae*, genome, the first for a chelicerate species and providing proof-of-concept that CRISPR-Cas9 can be used to create gene knockouts in mites (Dermauw et al., 2020). Development of such methods in ticks that allow gene knockout, knock-in, and gene replacement, is urgently needed to facilitate an understanding of tick genetics, biochemistry, development, and behavior. Additionally, the development of effective and reliable ancillary methods such as identification of promoters, transformation markers, embryology, and embryo injection, are needed for effective germline transformation. Here, we are reviewing the use of CRISPR/Cas, the most promising gene-editing approach, for tick genetic transformation and the ancillary methods needed for transgenic tick development.

## Current Research and Future Development Towards Tick Genetic Manipulation

### Ancillary Methods for Transgenesis

#### Transformation Markers

Screening for mutants is a challenge for non-model organism transgenesis. In most arthropods there is an absence of mutant strains and corresponding rescue transgenes, as in the white-eye gene of *Drosophila melanogaster* (Rubin and Spradling, 1982). Instead, fluorescent markers such as enhanced green fluorescent protein (EGFP), are frequently used as reporters for gene insertion and expression (Berghammer et al., 1999; Horn et al., 2000; Horn et al., 2002; Thomas et al., 2002) and may be useful tools for tick transformation.

##### 3xP3–EGFP as a Universal Transformation Marker for Arthropod Transgenesis

Evolutionarily conserved genetic circuitry governs all metazoan animal eye development under the control of the transcriptional activator Pax-6/Eyeless (Callaerts et al., 1997). Pax-6 homodimer binding site (P3) multimerization was shown to mediate photoreceptor-specific gene expression in *D. melanogaster* (Sheng et al., 1997). These studies led to the development of an artificial promoter, 3xP3. 3xP3 has been employed to drive robust and eye-specific expression of EGFP (Berghammer et al., 1999; Horn et al., 2000) and identify transgenic individuals in several different insect orders. The artificial 3xP3 promoter construct mediates EGFP expression in the eyes of insects and, like the constitutive promoters mentioned below, can be used to identify transgenic organisms at all stages: larval, pupal, and adult (Horn et al., 2000). The evolutionary conserved function of Pax-6 in eye development of metazoans (Callaerts et al., 1997) suggests that the 3xP3–EGFP marker should apply to all eye-bearing animals. In mosquitoes, 3xP3, expresses in eyes and ventral nerve cord (Volohonsky et al., 2017). An ortholog of *D. melanogaster* Pax-6 was also identified in the *I. scapularis* genome (ISCW003096/EEC03577) as well as other ticks such as *Rhipicephalus sanguines* (XM_037656705.1/XP_037512638) and *Dermacentor silvarum* (XM_037709500I/XP_037565428). It is therefore predicted that the 3xP3-EGFP marker should work in ticks that have well-developed eyes (such as *Ambylomma*, *Dermacentor*, and *Rhipicephalus*) and may have expression elsewhere in eyeless ticks of the genera *Ixodes* and *Haemaphysalis* (such as in the synganglion). Thus, 3xP3 with a tick-specific core promoter needs to be tested in ticks and new promoter/reporter systems need to be identified and developed.

##### Other Visible Markers

A transformation marker system that results in phenotypes visible to the naked eye due to changes in the color of melanin pigments is highly desirable for tick transgenesis. The final step of enzymatically-regulated melanin biogenesis is the conversion of dopachrome into dihydroxyindoles, a reaction catalyzed by a class of enzymes called dopachrome tautomerases (DCT) and the functionally redundant enzyme in insects, bacteria, and fungi, dopachrome converting enzyme/yellow protein (DCE) (Rosani et al., 2019). Yellow protein marker has been effectively utilized in mosquito transgenics (Li et al., 2017a). We identified an ortholog of DCT in the *I. scapularis* genome (ISCW009232) that may have similar melanin biogenesis functions and needs to be tested. Unfortunately, not all markers from other arthropods may be suitable for ticks. For instance, overexpression or knockout of Arylalkylamine-N-acetyl transferase and β-alanyl-dopamine synthetase (Bm-ebony) lightens coloration in *Bombyx mori*, *Harmonia axyridis* and *D. melanogaster* (Osanai-Futahashi et al., 2012), but their orthologs in *I. scapularis* genome have not been found. However, markers from more closely related arthropods may offer more promise. Mutations in a gene encoding a phytoene desaturase (tetur01g11270) were shown to cause an albino phenotype in *T. utricae* (lack of red pigment in the front legs and eyes) (Bryon et al., 2017). Knockout screening was enabled in this mite by injecting sgRNA and Cas9 into unfertilized females which produce only haploid male progeny that were easily screened for the albino phenotype (Dermauw et al., 2020). Although the orthologous sequences have not been identified in any tick genome, a similar gene product will be a promising target to develop for tick markers.

#### Promoters

Constitutive promoters allow the detection of transformants at all life stages. The polyubiquitin promoter was successfully utilized to generate the transformation marker PUbnlsEGFP in *D. melanogaster* (Handler and Harrell, 1999), and later to identify transformants in the Caribbean fruit fly, *Anastrepha suspensa* (Handler and Harrell, 2001), the Australian sheep blowfly, *Lucilia cuprina* (Heinrich et al., 2002), and mosquitoes (Anderson et al., 2010). Actin5C derived from *D. melanogaster* is another commonly used constitutive promoter to drive EGFP. The actin5C promoter works well at all developmental stages of *D. melanogaster* and the mosquitoes *Aedes aegypti*, *Anopheles stephensi*, and *Culex quinquefasciatus* (Catteruccia et al., 2000; (Pinkerton et al., 2000; Allen et al., 2001) but mediates only low and non-uniform EGFP expression levels in the stable fly, *Stomoxys calcitrans* (O’Brochta et al., 2000), suggesting that it is not broadly useful for all insects. Other promoters from *D. melanogaster* genes such as HSP70 and HSP82 have also been successfully used to drive marker genes in a variety of insects. While these promoters are widely useful, they are not universal. Transformation markers based on constitutive promoters have only been applied to closely related species, mostly within Diptera. Whether any such promoter can be functional across a wide range of insect orders, let alone more distantly related arthropods, remains to be tested.

Mammalian promoters with a GFP reporter, e.g. Cytomegalovirus immediate early promoter, human elongation factor 1a promoter, Human Ubiquitin C promoter, and chicken β-Actin promoter coupled with CMV early enhancer (CAGG), drive gene expression in *Drosophila* cell lines (Qin et al., 2010) and may be more broadly useful. Encouragingly, CAGG mCherry promoter was used to successfully transform an *I. scapularis* cell line, ISE6 (Oliver et al., 2015), yet it remains to be tested in tick embryos.

In order for work on tick transformation to proceed, endogenous tick promoters that work in a variety of tick species need to be identified. At the very least, a constitutive promoter that allows immediate identification of mutants after hatching is needed. Moving forward, tissue-specific or inducible promoters (such as the mosquito vitellogenin promoter that is fat body-specific and is activated after blood-feeding) would permit further refinement in tick transgenics. The availability of tick cell lines (Kurtti et al., 2008) provides an excellent tool for easily testing promoters *in vitro* before more challenging *in vivo* validation by embryo injections.

#### Early Embryonic Development

For the generation of successful stable mutants by embryo injection, early embryonic events such as the timing of cellularization and gonadal cell formation are essential to understand. This information is lacking in ticks and needs to be determined for efficient transgenic protocol development using CRISPR-Cas9 for knockout and knock-ins or classical transgenics using transposable elements.

Development of an embryo injection procedure requires knowledge of early embryonic development, including information about the timing of cellularization, anterior/posterior axis, and gonadal cell formation (pole cells in insects), but is currently lacking in ticks. For instance, only a handful of papers have observed embryogenesis in Ixodid (hard) ticks (Santos et al., 2013; Friesen et al., 2016), and none in the genus *Ixodes*. Even among these studies, early mitotic divisions were not examined because the earliest embryos used were ~24 h post egg laying in both *Rhipicephalus microplus* (Santos et al., 2013) and *Dermacentor andersoni* (Friesen et al., 2016). At 24 h post egg laying, the embryos were already near the fifth mitotic division and the nuclei were located in the periphery of the egg (Santos et al., 2013; Friesen et al., 2016). Based on these early divisions, Friesen et al. (2016) suggested that if nuclear division occurs at a constant rate, the post-oviposition mitotic division rate in *D. andersoni* will be every 5 h. This nuclear division rate is much slower than described in *Drosophila melanogaster*, where early mitotic divisions occur as fast as every 8 min (Foe and Alberts, 1983; Gilbert, 2000). In *D. melanogaster* embryos, the first 13 nuclear divisions occur without cytokinesis, resulting in the syncytial blastoderm. Whether or not the early mitotic divisions in tick embryos are holoblastic (with mitosis and cytokinesis) or syncytial remains to be confirmed (Fagotto et al., 1988; Campos et al., 2006; Santos et al., 2013; Friesen et al., 2016). *Ixodes* ticks have a longer embryogenesis time (~40 days compared to ~11 days of *R. microplus*) suggesting that the early embryonic events will be delayed in these ticks. Understanding these embryonic development events in ticks would facilitate efficient transgenesis by embryo injection.

### Strategies for Tick Gene-Editing

#### Genetic Manipulation by Injecting CRISPR/Cas9 Components in Embryos

**Figure 1:**
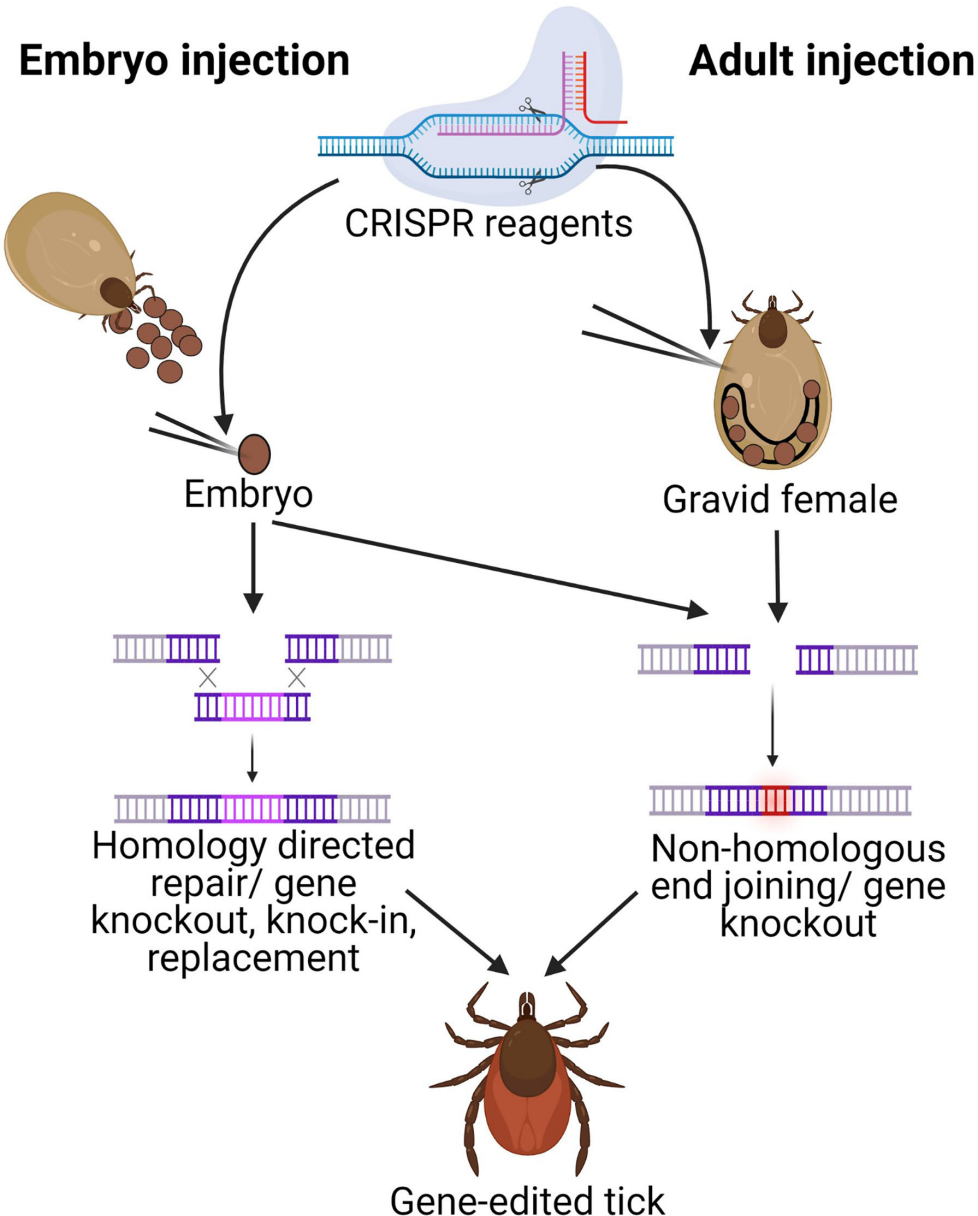
Schematic of gene-editing in ticks by injecting either embryos or gravid adults. CRISPR reagents are injected either into newly deposited embryos (embryo injection) or into gravid females (adult injection). Adult injections require ReMOT components (ligand attached to Cas9 protein and endosomal escape reagent) in addition to sgRNA for delivery to the egg. Both techniques utilize Cas9 to induce a dsDNA break at the sgRNA site.The cell’s repair machinery can introduce mutations through non-homologous end joining (NHEJ) in either technique for gene knockouts, but only embryo injections can currently introduce plasmids with homology arms necessary for homology directed repair (HDR) for gene knock-in. Image generated in Biorender.com.

Microinjection into newly deposited arthropod eggs (embryos) allows modification of the embryonic germline before it has differentiated (prior to cellularization), ensuring a heritable modification (**Figure 1**). Although approaches such as the gene gun (Thomas et al., 2001) and electroporation (Kamadar et al., 1992) have been tested in arthropods, embryo microinjection remains the most common approach for delivering gene-editing tools to the nucleus for genome modification. An embryo injection protocol was first established for the genetic transformation of *D. melanogaster* using transposable elements. This classical protocol (Rubin and Spradling, 1982) has been adapted to allow the injection of various types of nucleic acid constructs such as transformation vectors (P-elements, Piggybac, Hermes, etc.) and their helper plasmids, RNA, single guide RNA (sgRNA), plasmids, and Cas9 mRNA (or Cas9 protein).

The foundational work to develop injection methods has made the generation of transgenic *Drosophila* and even mosquitoes (*Aedes* and *Anopheles*) a routine laboratory procedure. Details about the physical operations involved in producing transformants such as DNA preparation, eggs, needles, and injections have been thoroughly described for these insects (Kiehart et al., 2007; Kistler et al., 2015). The general procedure involves collecting freshly laid eggs and dechorionating them using diluted bleach (Iwamatsu, 1983). The eggs are then attached to a glass coverslip with double-sided tape and slightly desiccated to make room for the injection volume. Preblastoderm stage eggs are then injected at their posterior end (before pole cell formation) with the injection mix. The resulting adults (G_0_) are backcrossed and subsequent progeny are screened for the presence of the marker gene.

We recently developed an embryo injection protocol for *I. scapularis* and provided proof-of-principle for gene-editing in ticks. (Sharma et al., 2020, preprint). While early embryology in ticks is still not completely understood, our preliminary work has opened the door for further refinement of the injection protocol and resulting gene-editing methods. However, heritable germline mutations in ticks remain to be demonstrated. We expect that development of the above-mentioned ancillary methods coupled with our embryo injection protocol will make the generation of transgenic ticks a routine laboratory procedure, similar to mosquitoes.

#### Genetic Manipulation by Injecting CRISPR/Cas9 Components in Gravid Females

Despite our recent success at gene-editing ticks, tick embryo microinjection remains technically challenging. Two alternative methods that bypass the requirement for embryonic microinjection have been developed for insects: 1. Receptor‐Mediated Ovary Transduction of Cargo (ReMOT) and 2) Branched Amphiphilic Peptide Capsules (BAPC)- assisted CRISPR delivery (Chaverra-Rodriguez et al., 2018; Hunter et al., 2018) (**Figure 1**). ReMOT is based on delivery of the CRISPR/Cas9 ribonucleoprotein (RNP) complex (Cas9 with an sgRNA) by using peptide ligands derived from *D. melanogaster* yolk protein precursors (YPPs) fused to the Cas9‐RNP complex. Chemical compounds such as chloroquine or saponin are used as endosomal escape reagents (EER) that facilitate the escape of the YPP‐RNP complex from endosomes into the oocyte cytoplasm. The injection of the YPP‐RNP/EER complex into the hemolymph of vitellogenic females of several insect species enabled targeted gene editing in embryos (Chaverra-Rodriguez et al., 2018; Heu et al., 2020; Macias et al., 2020; Shirai and Daimon, 2020). The BAPC‐assisted CRISPR delivery involves the use of branched amphiphilic peptide capsules BAPtofect™ (Phoreus™ Biotechnology, Inc. Olathe, Kansas, US) for delivery of CRISPR RNP into the ovary (Sukthankar et al., 2014) and was recently used to improve the delivery of CRISPR components into ovaries of the adult Asian citrus psyllid, *Diaphorina citri* (Hunter et al., 2018) to facilitate heritable gene editing.

Our recent work successfully utilized ReMOT technology for gene editing in *I. scapularis* suggesting promise for this technique as a straightforward method for gene knockout in ticks (Sharma et al., 2020, preprint). The ReMOT reagents were stable in ticks for a maximum of 48 h, necessitating multiple injections during the relatively long egg maturation and deposition time in ticks. BAPC-assisted CRISPR delivery has yet to be tested in ticks, but this method relies on stable peptide capsules which may have improved stability and avoid the need for multiple hemolymph injections. However, a limitation of both of these alternate strategies is that they cannot currently be used for gene knock-in (overexpression or replacement) as there is no mechanism for carrying template into the embryo for homology-directed repair, making them suitable only for gene knockout studies.

## Discussion

The availability of tick genome sequences (Cramaro et al., 2015; Gulia-Nuss et al., 2016; Cramaro et al., 2017; Nuss et al., 2018; Jia et al., 2020) have already made application of molecular methods possible in ticks. However, study of the molecular biology of ticks is currently limited by the applicability of genetic tools. The need to achieve efficient methods for germline transformation in ticks remains a high priority for functional genomics research. For this to happen, the groundwork needs to be built for ancillary methods such as embryology, embryo injection, and identification of tick promoters and markers. Embryo injection methods are crucial for the development of transgenic lines and necessary for other applications such as stable infection with symbionts. Similarly, the development of gene-editing methods that do not require embryo injection (such as ReMOT) can be used for gene knockouts to help make genetically engineered ticks a common lab practice. Additional refinement of this method may permit gene knock-ins to be used for different applications such as gene replacement and over-expression.

CRISPR/Cas technologies have made targeted genetic engineering feasible in most organisms. Proof-of-principle experiments in *Ixodes* demonstrate that this technique is feasible in ticks as well (Sharma et al., 2020 preprint). However, stable germline transformation in ticks has not yet been confirmed. Therefore, a future focus should be on creating stable lines for fundamental research. Beyond a fundamental understanding of tick biology, a further application of tick genetic transformation is population control by inserting gene drives. Gene drives, which bias inheritance towards a natural or synthetic genetic element or specific allele and lead to a preferential increase of a specific phenotype throughout a population (Alphey et al., 2020), are being developed for mosquito control. Several different gene drives, autonomous/non-autonomous, split, self-limiting drives, etc., have been developed (Gantz et al., 2015; Hammond et al., 2016; Patil et al., 2018; Adolfi et al., 2020; Carballar-Lejarazú et al., 2020; Li et al., 2020). For instance, a male dominant allele to produce a single sex to reduce tick populations, or a trait to increase refractoriness to pathogens, could be effective strategies for managing tick-borne diseases.

Further research into the areas identified in this opinion piece will provide the necessary steps to develop routine genetic transformations in ticks. Once this is possible, it will provide a badly needed set of tools to understand gene function in ticks. This will open the door to understanding a group of truly unique arthropods and how we might manage these major vectors of human and animal disease.

## Data Availability Statement

The original contributions presented in the study are included in the article/supplementary material. Further inquiries can be directed to the corresponding author.

## Author Contributions

AN, AS, and MG-N wrote the initial drafts. MG-N and AN wrote the final draft. All authors contributed to the article and approved the submitted version.

## Funding

This work was funded by NIH-NIAID R21AI128393 and Plymouth Hill Foundation, NY to MG-N, Startup funds from the University of Nevada to AN, Peer-to-Peer Grant from IGTRCN to AS.

## Conflict of Interest

The authors declare that the research was conducted in the absence of any commercial or financial relationships that could be construed as a potential conflict of interest.
